# Absence of Transport Altermagnetic Spin-Splitting Effect in RuO_2_

**DOI:** 10.1021/acs.nanolett.5c05787

**Published:** 2026-01-29

**Authors:** Yu-Chun Wang, Zhe-Yu Shen, Chia-Hsi Lin, Wei-Chih Hsu, You-Sheng Chen, Yi-Ying Chin, Akhilesh Kr. Singh, Wei-Li Lee, Chien-Te Chen, Ssu-Yen Huang, Danru Qu

**Affiliations:** † Department of Physics, 34879 National Taiwan University, Taipei 10617, Taiwan; ‡ Center for Condensed Matter Sciences, National Taiwan University, Taipei 10617, Taiwan; § Department of Physics, National Chung Cheng University, Chia-Yi 621301, Taiwan; ∥ 71551 Institute of Physics, Academia Sinica, Taipei, 115201, Taiwan; ⊥ 57815 National Synchrotron Radiation Research Center, Hsinchu 300092, Taiwan; # Center of Atomic Initiatives for New Materials, National Taiwan University, Taipei 10617, Taiwan

**Keywords:** spin current, spintronics, altermagnetism

## Abstract

Altermagnets have attracted significant interest recently. Through the altermagnetic spin-splitting effect (ASSE), a longitudinal spin-polarized or a transverse pure spin current can be generated upon charge current injection. The ASSE is a key experimental feature for altermagnets but is often mixed with the spin Hall effect (SHE). Here, we present a comprehensive study of spin-to-charge conversion in epitaxial ruthenium dioxide (RuO_2_) thin films using the ferromagnetic insulator yttrium iron garnet (YIG) as the spin current source. We conclusively show the absence of the ASSE in RuO_2_ films grown with three different crystal orientations. Instead, we attribute the spin-to-charge conversion signals solely to the SHE. Moreover, we reveal a negative spin Hall angle in RuO_2_ when it is adjacent to YIG, which reverses the sign when interfaced with Py. Our study provides crucial insights into the recent arguments on RuO_2_ and advances the understanding of spin-to-charge conversion in low-symmetry materials.

## Graphical abstract

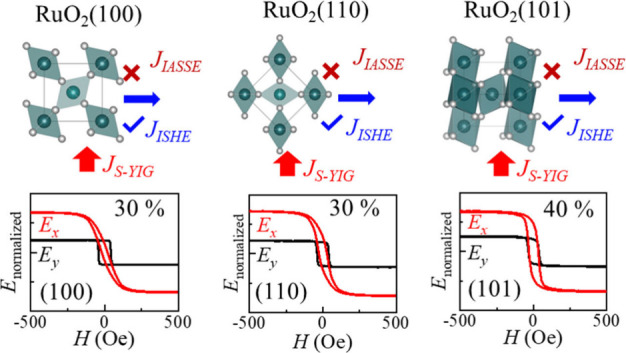

Altermagnetism has recently garnered significant attention as a new category of magnetism, alongside ferromagnetism and antiferromagnetism.^1,2^ One prototypical candidate material is ruthenium dioxide (RuO_2_),^3^ which has a rutile crystal structure with space group number 136 (*P*4_2_/*mnm*) and lattice constants of *a* = *b* = 4.5 Å and *c* = 3.1 Å. Early spectroscopic studies, including neutron diffraction^4^ and resonant X-ray scattering,^5^ revealed antiferromagnetic order in RuO_2_ with Néel vectors aligned along the [001] direction. Thus, with the *C*_2_ magnetic and *C*_4_ crystallographic symmetries, RuO_2_ serves as an ideal metallic *d*-wave altermagnetic candidate.^2^ However, recent spectroscopic investigations using muon spin resonance,^6^ neutron scattering,^7^ and spin- and angle-resolved photoemission spectroscopy^8^ have reported the absence of magnetic order RuO_2_, casting serious doubt on the existence of altermagnetism in this material.

On the other hand, a key advantage of altermagnetic materials is the ability to generate spin currents through nonrelativistic spin-splitting effects. Accordingly, investigating and understanding the spin-dependent transport in altermagnetic candidates are crucial for spintronic applications. Pioneering studies in RuO_2_ have revealed pronounced anisotropic or unconventional spin-charge interconversion.^9–14^ Such anisotropic or unconventional spin accumulations are often interpreted as signatures of the altermagnetic spin-splitting effect (ASSE) or its inverse effect (IASSE), which are considered transport hallmarks of altermagnetism. However, recent spin pumping^15^ and terahertz emission^16^ experiments suggest that the relativistic spin Hall effect (SHE), instead of the ASSE, dominates the transport behaviors in RuO_2_. More recent spin-splitting torque^17^ and spin-splitting magnetoresistance measurements,^18,19^ however, again consider significant contributions from ASSE-induced spin currents. The controversy in the transport study of RuO_2_ is still not conclusively settled. Since most altermagnetic candidates, including RuO_2_, inherently possess low crystal symmetry,^2^ that is, they are either noncubic or have a space group number below 200, a comprehensive understanding of their spin-dependent transport properties is essential for disentangling the origin of the anisotropic and unconventional responses (see Supporting Information S1).

In this work, we provide a comprehensive study of the spin-to-charge conversion in RuO_2_. Significantly different from previous reports, we use a ferromagnetic *insulator* yttrium iron garnet (YIG) as a capping layer to eliminate charge current complications and to supply spin current into epitaxially grown RuO_2_ via the spin Seebeck effect (SSE). To provide a complete comparison, we adopt *three* widely established thin-film deposition techniques: magnetron sputtering, oxide molecular beam epitaxy (oxide MBE), and pulsed laser deposition (PLD) to deposit high-quality epitaxial RuO_2_ films on *three* crystal orientations of TiO_2_ substrates, namely, the (100)-, (110)-, and (101)-orientations. Remarkably, we observe robust and anisotropic spin-to-charge conversion in RuO_2_ regardless of the deposition method and crystal orientations. Through careful analysis, we conclusively show the absence of the ASSE and the dominance of the SHE in all of these RuO_2_ thin films. We obtain directly from the experiments the three independent spin Hall conductivities (σ_SH_) and spin Hall angle (*θ*_SH_) tensor components for RuO_2_. Moreover, across the three deposition methods, we consistently observe *negative θ*_SH_ for RuO_2_ films when they are next to YIG, which are *opposite* in sign to all the previous reports using Py/RuO_2_. Our findings provide critical insight into the recent arguments regarding RuO_2_ and offer a framework for studying other altermagnet candidates with low crystalline symmetry.

We begin by illustrating the transverse spin-to-charge conversion in an ideal *d*-wave altermagnet with Néel vectors (*N*) aligned along the [001]-direction. The *d*-wave spin-splitting bands are depicted in Figures 1a-d, where the solid blue and red ellipses represent the energy bands for opposite spin states, as indicated by the arrowheads and tails. When a spin current (*J*_S_) is injected along the [100]-direction, with the spin polarization (σ) pointing along the [001]-direction, as illustrated in Figure 1a, the shift of the *d*-wave spin bands (marked by the dotted lines and shaded areas) gives rise to a transverse charge current along the [010]-direction via the IASSE, denoted as *J*_IASSE_. In contrast, when *J*_S_ is injected along the [110]-direction, or when σ is oriented perpendicular to [001], as illustrated in Figures 1b-d, no *J*_IASSE_ is generated.

**Figure 1:**
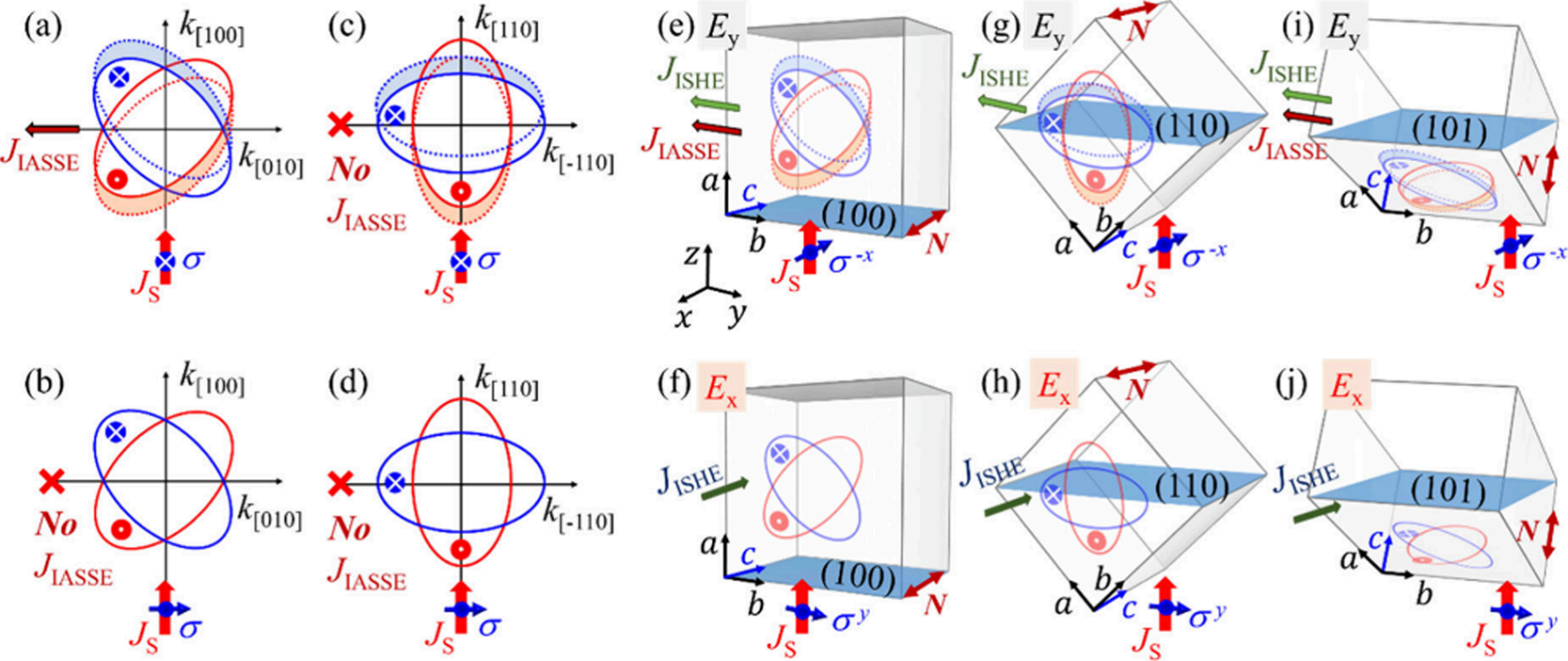
A spin current *J*_S_ (red arrow) is injected into the *d*-wave spin-splitting bands (red and blue ellipses) along the (a) [100]-, (b) [100]-, (c) [110]-, and (d) [110]-directions, with spin polarization σ aligned along the (a) [001]-, (b) [010]-, (c) [001]-, and (d) [110]-directions, respectively. Arrow tails and heads denote opposite spin states. Solid and dotted ellipses represent the spin-splitting bands before and after spin current injection. The shifted areas of the Fermi surface are shaded in blue and red. Only in panel a is a transverse charge current induced by the IASSE *J*_IASSE_ (dark red arrow). Panels e-j illustrate simplified tetragonal unit cells of rutile RuO_2_, with shaded blue areas representing the (100)-, (110)-, and (101)- crystallographic planes, lying in the *xy*-plane of the measurement coordinate system. The [100]-, [010]-, and [001]-directions are labeled *a*, *b*, and *c*, respectively. The Néel vector *N* (double arrow) is aligned along the *c*-axis. A spin current is injected along the *z*-axis into the three crystal cuts, with spins aligned along the -*x*- and *y*-axes. A transverse *J*_IASSE_ is generated only in the (100)- and (101)-samples in e) and (i), but not for the (110)-sample in g), whereas *J*_ISHE_ is present in all cases.

As further illustrated in Figure 1e–j, we examine three crystal orientations of RuO_2_, the (100)-, (110)-, and (101)-planes, which lie in the *xy*-plane of the measurement coordinate system. A spin current, *J*_S_ is injected vertically into these planes along the *z*-axis. The resulting electromotive force along the *y*-axis is denoted as *E*_*y*_ [Figure 1e, g, and i], while that along the *x*-axis is denoted as *E*_*x*_ [Figure 1f, h, and j]. Assuming RuO_2_ is a *d*-wave altermagnet with *N* along the *c*-axis, then when σ is aligned with the -*x*-axis, as shown in Figure 1e, g, and i, only for the (100)- and (101)-, but *not* the (110)-planes, a transverse *J*_IASSE_ is induced. When σ is oriented along the *y*-axis, which is perpendicular to *N*, as depicted in Figure 1f, h, and j, no *E*_*x*_ is generated by the IASSE in any of the orientations.

Additionally, an inverse spin Hall effect (ISHE), with an induced charge current *J*_ISHE_, is expected in all cases shown in Figure 1e–j, due to the sizable spin–orbit coupling in RuO_2_. Symmetry analysis of the rutile structure with space group No. 136 reveals the coexistence of three independent σ_SH_,^20^ which could result in anisotropic spin-to-charge conversion in RuO_2_. For both the (100)- and (101)-planes, the IASSE and ISHE are mixed and inseparable. But for the (110)-plane, as shown in Figure 1g–h, regardless of the spin orientation, no transverse IASSE is induced. Hence, the (110)-plane plays a crucial role in revealing the anisotropic SHE in RuO_2_ and is key to unambiguously distinguishing between anisotropic ISHE and IASSE experimentally.

The RuO_2_ layers studied in this work are fabricated on TiO_2_ substrates with three different crystal orientations, the (100)-, (110)-, and (101)-orientations, using DC sputtering at an elevated temperature of 500 °C. To further understand the influence of fabrication methods on the observed signals, we prepare reference RuO_2_ samples using oxide-MBE at 350 °C and PLD at 650 °C. These samples are denoted as RuO_2_^S^, RuO_2_^M^, and RuO_2_^P^, corresponding to sputtering, MBE, and PLD growth, respectively. The YIG layer is deposited onto the RuO_2_ layer by radio frequency (RF) sputtering at room temperature, followed by rapid thermal annealing in an oxygen atmosphere at 800 °C. Magnetization measurements show that after annealing, the YIG layer crystallizes with sizable magnetization [see Supporting Information S2]. We confirm the epitaxial relationship between the RuO_2_ layer and the TiO_2_ substrate using both X-ray diffraction spectroscopy (XRD) and transmission electron microscopy (TEM) [see Supporting Information S3]. For comparison, we also prepare a reference Pt sample, with the Pt film deposited onto the epitaxial YIG film grown on the (111)-oriented gadolinium gallium garnet (GGG) substrate, [see Supporting Information S4], as well as a reference permalloy (Py) sample, with the Py film deposited directly onto the epitaxial RuO_2_ film. All measurements in this work are conducted at room temperature.

We first perform spin Seebeck measurements (SSE) to capture the anisotropic spin-to-charge conversion in RuO_2_. As shown in Figures 2a-b, under a vertical temperature gradient of *∇T* = 13 K mm^–1^, a magnon spin current is driven along the *z*-axis in YIG^21^ and injected vertically into the underlying RuO_2_ layer. The spin current is subsequently converted into a transverse charge current via IASSE or ISHE. The resulting charge accumulation is directly detected as a voltage (*V*) in the RuO_2_ layer. We estimate the electromotive force (*E*) using *E* = *V*/*d*, where *d* is the distance between the electrodes. The electromotive force obtained along the *x*- and *y*-axes is denoted as *E*_*x*_ and *E*_*y*_, corresponding to those illustrated in Figures 1e-j. For the magnetic field (*H*) angular-dependent measurements, *H* is rotated within the *xy* plane, with the angle ϕ defined relative to the *x*-axis. The *E*_*y*_/*E*_*x*_ ratio nicely captures the anisotropy of the spin-to-charge conversion in RuO_2_.

**Figure 2:**
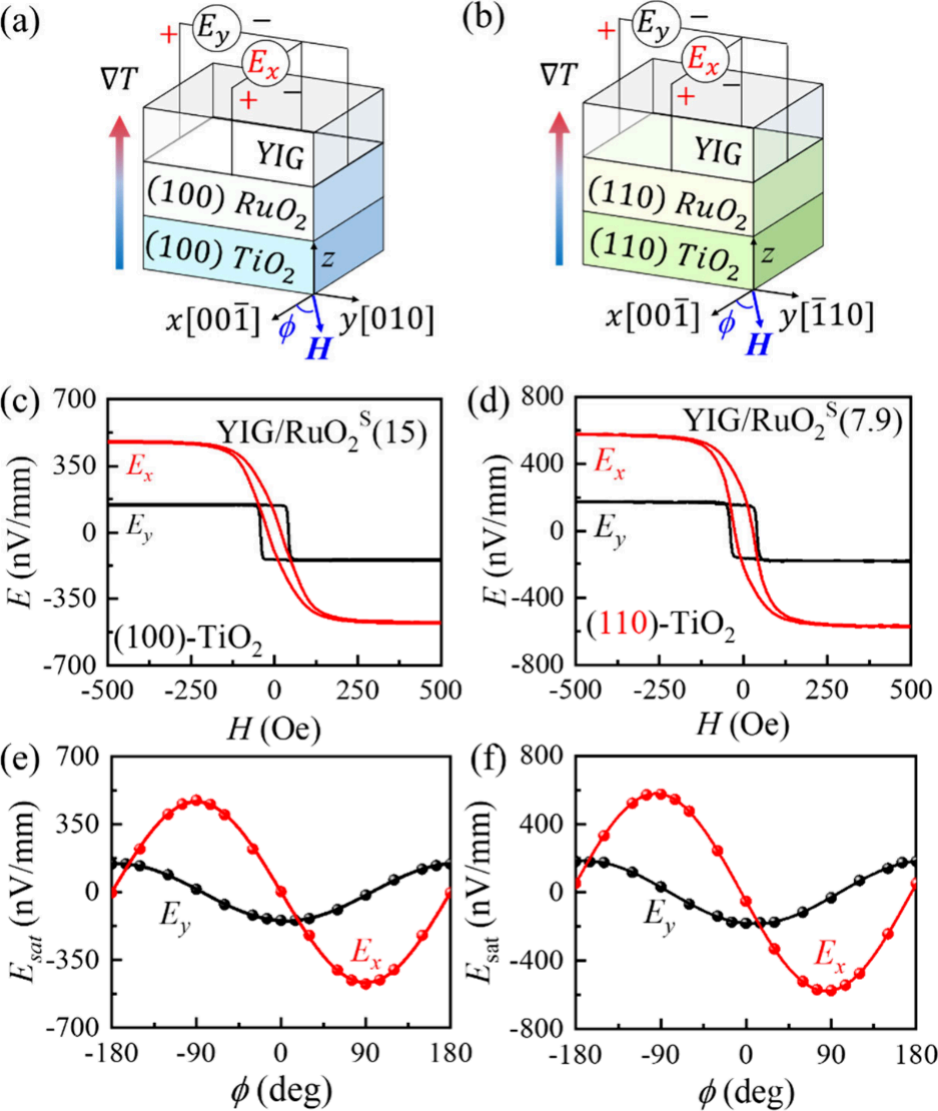
Schematic illustrations of the experimental setup for a) (100)- and b) (110)-oriented YIG/RuO_2_/TiO_2_ samples. The *x*- and *y*-axes are aligned with the a) [001]- and [010]-, and b) [001]- and [110]-crystallographic directions, respectively. The bonding wires directly contact the RuO_2_ layer for the voltage measurements. The angle ϕ denotes the orientation of the external magnetic field *H* relative to the *x*-axis. The spin Seebeck voltage and *H*-angular dependence are measured for the sputter-fabricated c), e) (100)-RuO_2_ and d), f) (110)-RuO_2_ samples.

As shown in Figure 2a, for the 15 nm-thick (100)-oriented RuO_2_ film, a spin current is generated and flows along the *z*-axis, i.e., the [100]-direction. With spins aligned parallel (along the *x*-axis) and perpendicular (along the *y*-axis) to the [001]-direction, we observe induced electromotive forces of *E*_*y*_ = −145 nV mm^–1^ and *E*_*x*_ = −473 nV mm^–1^, respectively, as shown in Figure 2c. The *E*_*y*_/*E*_*x*_ ratio is approximately 30%, consistent with that obtained in our previous work.^9^ According to Figures 1e and f, *E*_*y*_ contains contributions from both ISHE and IASSE, whereas *E*_*x*_ arises solely from ISHE. Therefore, if the IASSE significantly contributes to the anisotropic spin-to-charge conversion, the voltage ratio for other crystalline orientations, particularly the (110)-oriented RuO_2_, which has *no* transverse ASSE contribution at all, must be sharply different.

However, as we demonstrated in Figure 2d, for the (110)-oriented RuO_2_, where the spin current is injected along the [110]-direction and the spin is aligned along the [001]- or [110]-directions, the voltage signals still exhibit considerable anisotropy with *E*_*y*_ = −181 nV mm^–1^ and *E*_*x*_ = −574 nV mm^–1^, revealing an *E*_*y*_/*E*_*x*_ ratio of 30%, nearly *identical* to that observed in the (100)-plane. Without any IASSE contribution, the anisotropic voltage observed in (110)-RuO_2_ is *solely* from the ISHE.

From a symmetry point of view,^20^ RuO_2_ with space group No. 136 (*P*4_2_/*mnm*) supports three independent spin Hall conductivity (SHC) tensor components, denoted as σ_*ab*_^*c*^ = −σ_*ba*_^*c*^ = *A*, σ_*bc*_^a^ = −σ_*ac*_^*b*^ = *B*, and σ_*ca*_^*b*^ = -σ_*cb*_^*a*^ = *C*, where *a*, *b*, and *c* correspond to the [100] -, [010] -, and [001]- crystal directions, respectively. The altermagnetic spin-splitting conductivity is denoted as σ_ASSE_, which accounts for the spin-to-charge conversion via spin-splitting effects. Using a transformation matrix discussed in Supporting Information S5, we derive

1 $$E_{y}/{E_{x}}_{\left(100\right)}=\left(A+\sigma_{\mathrm{ASSE}}\right)/B$$

For the (100)-orientation and,

2 $$E_{y}/{E_{x}}_{\left(110\right)}=A/B$$

for (110)-orientation.

The nearly identical 30% ratio for both (100)- and (110)-oriented RuO_2_ suggests

3 $$\left(A+\sigma_{\mathrm{ASSE}}\right)/B\approx A/B\approx 30\%$$

which conclusively reveals that σ_ASSE_ ∼ 0. The altermagnetic spin-splitting contribution σ_ASSE_ is *absent* in the RuO_2_ that we studied. The observed voltage anisotropy arises *entirely* from the anisotropic spin Hall conductivity, where A/B ≈ 30%.

As a comparison, for the (101)-oriented film, as illustrated in Figure 3a, the spin current flows normal to the film surface, with spins oriented along the [101̅]- and [010]-directions. As shown in Figure 3c, we observe *E*_*y*_ = −253 nV mm^–1^ and *E*_*x*_ = −677 nV mm^–1^, yielding a slightly larger *E*_*y*_/*E*_*x*_ ratio of about 40%. Using

4 $${\frac{E_{y}}{E_{x}}}_{\left(101\right)}=\frac{\left(A+\sigma_{ASSE}\right)\sin^{2}\left(\theta_{c}\right)+C\cos^{2}\left(\theta_{c}\right)}{C\cos^{2}\left(\theta_{c}\right)+B\sin^{2}\left(\theta_{c}\right)}$$

from Supporting Information S5, where *θ*_*c*_ = 34.56° denotes the angle between the (001) and (101) planes, A/B≈ 30% and σ_ASSE_ ∼ 0, we can extrapolate the relative relationships between the three independent SHC tensors, and obtain C/B ≈ 8%. These results are summarized in Table 1. Notably, the magnetic field (*H*) angular-dependent measurements of *E*_*y*_ and *E*_*x*_, with *H* rotated in the *xy* plane, for the (100)-, (110)-. and (101)- planes, as illustrated in Figure 2e, f, and Figure 3e, are nicely fit by the cosine and sine functions, with minima at 0° and 90°, respectively.

**Figure 3:**
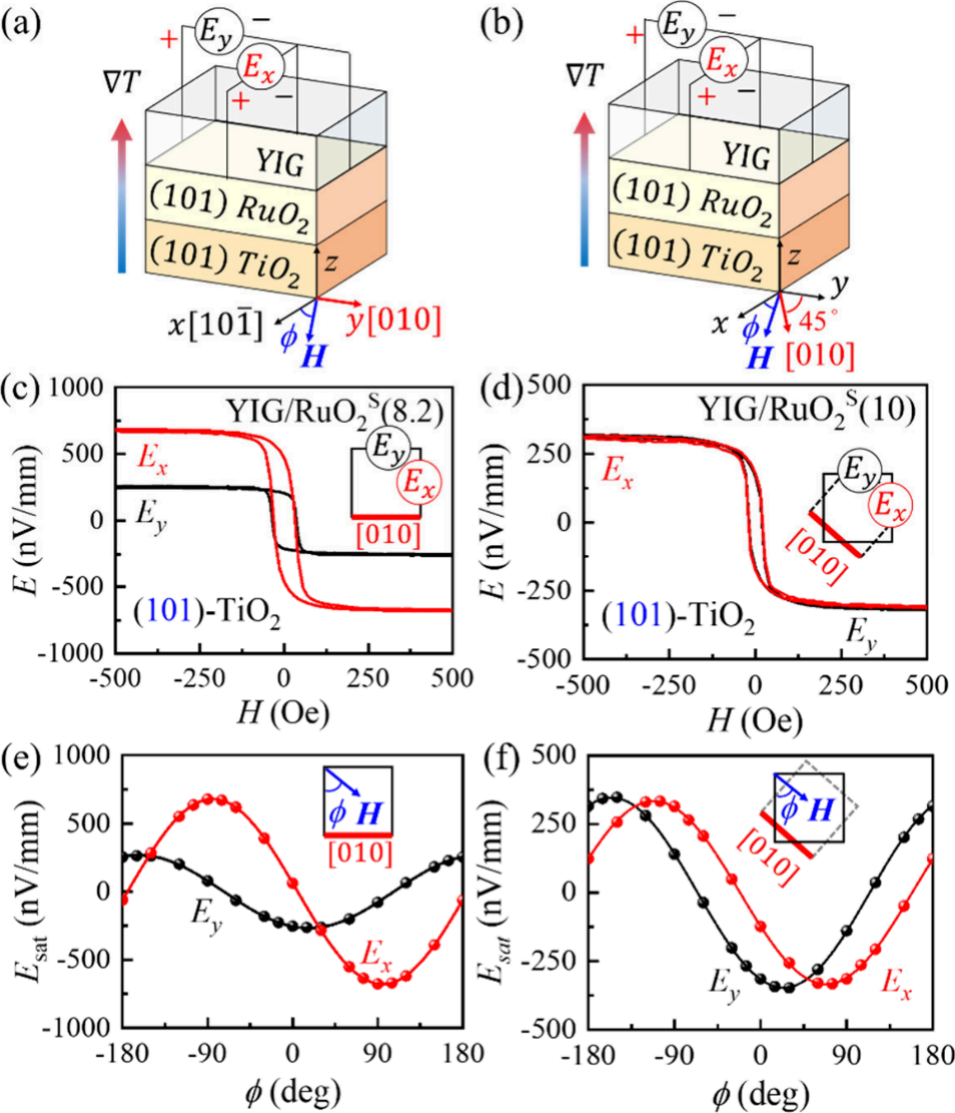
Schematic illustrations of the experimental setup for the (101)-oriented YIG/RuO_2_/TiO_2_ samples with different in-plane cuts, where the *y*-axis is a) aligned with the [010]-direction (denoted as regular cut) and b) rotated 45° counterclockwise from the [010]- direction (denoted as 45°-cut). The bonding wires directly contact the RuO_2_ layer for voltage measurements. The angle ϕ denotes the orientation of the external magnetic field *H* relative to the *x*-axis. The spin Seebeck voltage and *H*-angular dependence are measured for the sputter-fabricated (101)-RuO_2_ films with the (c, e) regular cut and (d, f) 45°-cut.

**Table 1:** Summary of the Anisotropic Spin Hall Angle (SHA), Spin Hall Conductivity (SHC), and Anisotropy Ratio Obtained from the Anisotropic Spin-to-Charge Conversion in RuO_2_

SHA (%)	${\theta_{SH}}_{bc}^{a}$	${\theta_{SH}}_{ca}^{b}$	${\theta_{SH}}_{ab}^{c}$
	–4.0 ± 0.8	–0.3 ± 0.06	–1.2 ± 0.2
SHC (S cm^–1^)	σ_*bc*_^*a*^ = −σ_*ac*_^*b*^	σ_*ca*_^*b*^ = −σ_*cb*_^*a*^	σ_*ab*_^*c*^ = −σ_*ba*_^*c*^
	–250 ± 51	–19 ± 3	–75 ± 15
Ratio (%)	σ_*bc*_^*a*^/σ_*bc*_^*a*^	σ_*ca*_^*b*^/σ_*bc*_^*a*^	σ_*ab*_^*c*^/σ_*bc*_^*a*^
	100	8	30

To further confirm the absence of ASSE in RuO_2_, we performed an additional measurement. We employ a different square cut (denoted as the 45°-cut) of the (101)-oriented sample, as shown in Figure 3b, where the new *x*- and *y*-axes are rotated 45° in-plane, counterclockwise from the original coordinates. For the new sample, using A/B = 30%, C/B = 8% and σ_ASSE_ = 0, we expect the voltage ratio *E*_*y*_/*E*_*x*_ to be 100%, and the voltage minimum to be located at 23° and 67°, respectively, for *E*_*y*_ and *E*_*x*__,_ as discussed in Supporting Information S6. Our experimental results in Figures 3d and 3f show excellent consistency with our prediction. These results further confirm the absence of the ASSE contribution.

For the (101)-plane, we also notice that the spin Hall conductivity tensor component σ_*zy*_^*z*^ is nonzero (see Supporting Information S6), indicating that a charge current along the *y*-axis generates an unconventional *z*-polarized spins that flow along the *z*-axis, in addition to the conventional *x*-polarized spins. Our calculations yield a *z*-spin to *x*-spin ratio of −0.675, corresponding to an effective spin moment tilted 34° off the *x*-axis toward the -*z*-axis. The *z*-spin can be further utilized to switch a perpendicular magnet.

To verify that the observed anisotropy is independent of the fabrication method and extrinsic impurities, we perform the spin Seebeck voltage measurements on RuO_2_ films grown via PLD and MBE. As shown in Figures 4a and b, consistent voltage anisotropy is observed. The *E*_*y*_/*E*_*x*_ ratios in these RuO_2_ films remain impressively ∼30% for the (100)-plane and ∼40% for the (101)-plane. Across a total of 15 samples fabricated by sputtering, PLD, and MBE, the averaged *E*_*y*_/*E*_*x*_ ratios are 31.9 ± 5.6%, 28.4 ± 4.1%, and 38.9 ± 5.2%, for (100)-, (110)-, and (101)-orientations, respectively, as summarized in Figure 4c. The error bars arise from the standard error of the slightly varying *E*_*y*_/*E*_*x*_ ratios for these samples. Importantly, unlike most studies that report anisotropy using different samples, our work extracts the spin Hall conductivity ratios A/B and C/B within the *same* sample. While the absolute values of A, B, and C may vary by sample, their relative ratios are intrinsic and reproducible, regardless of thickness and fabrication method. The consistency of the *E*_*y*_/*E*_*x*_ ratios across samples fabricated by different techniques and with varying thicknesses highlights the robustness of the anisotropic spin Hall effect in RuO_2_, which is governed by its rutile crystal symmetry. The results also consistently suggest the absence of the transport altermagnetic spin-splitting character in all of the RuO_2_ films we studied.

**Figure 4:**
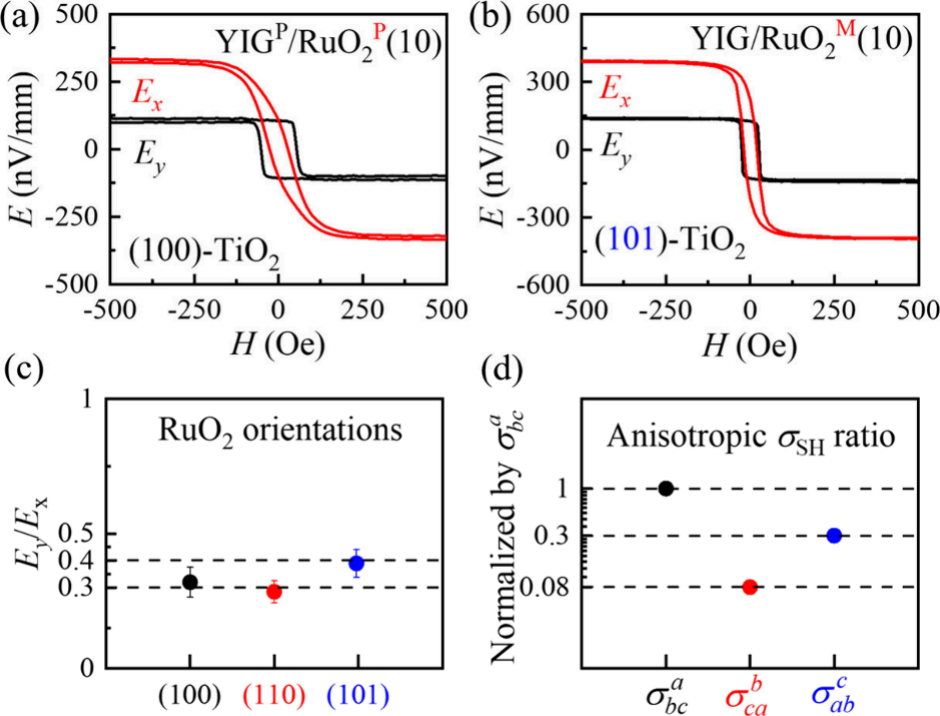
Anisotropic voltage signals measured in (a) PLD-fabricated RuO_2_^P^ and (b) MBE-fabricated RuO_2_^M^ samples. (c) A summary of orientation-dependent *E*_*y*_/*E*_*x*_ ratios for all 15 samples examined in this study. (d) Ratios of the anisotropic spin Hall conductivities among the three independent components: σ_*bc*_^*a*^, σ_*ca*_^*b*^, and σ_*ab*_^*c*^.

To understand the magnetic ground state of our RuO_2_, we perform two independent measurements, including magnetic field annealing of the YIG/RuO_2_/TiO_2_ sample and X-ray magnetic circular dichroism (XMCD) measurements (see Supporting Information S7–S8). The anisotropic voltage ratio remains unaltered before and after the field annealing process. The XMCD results also reveal no detectable magnetic signals. These results support the conclusion that RuO_2_ in our study is unlikely to be altermagnetic.

Furthermore, to provide a comprehensive study of the spin Hall effect in RuO_2_, we investigate its spin Hall angle tensor component $\left({\theta_{\mathrm{SH}}}_{jk}^{i}\right)$ , which is often simplified as a single value in other studies. Here, *i*, *j*, and *k* denote the direction of the spin, spin current, and charge current. To identify the sign of *θ*_SH_ for RuO_2_, we perform a direct comparison to that of Pt. In Pt, a positive thermal voltage *V* is observed along the + *x* direction when a temperature gradient *∇T* is applied along the + *z* direction and a magnetic field *H* is applied along the *x* + *y* direction, as shown in Figure 5b. The sizable electromotive force of *E* = 1635 nV mm^–1^, as shown in Figure 5d, corresponds to a positive *θ*_SH_ ≈ + 4% for Pt. By contrast, RuO_2_ under similar experimental conditions^22^ (Figure 5a and Supporting Information S9) exhibits a negative *θ*_SH_ throughout the measurements when YIG is used as a spin current source, as shown in Figure 5c. The negative *θ*_SH_ for YIG/RuO_2_ observed in our study is contrary to prior reports on RuO_2_ films in proximity to a ferromagnetic metal (FM) layer, such as Py (see Supporting Information S10), but is consistent with the negative *θ*_SH_ reported for annealed RuO_2_ grown on YIG.^23^ By further employing spin pumping measurements, we consistently demonstrate the *opposite* signs in *θ*_SH_ for YIG/RuO_2_ and Py/RuO_2_ (see Supporting Information S11).

**Figure 5:**
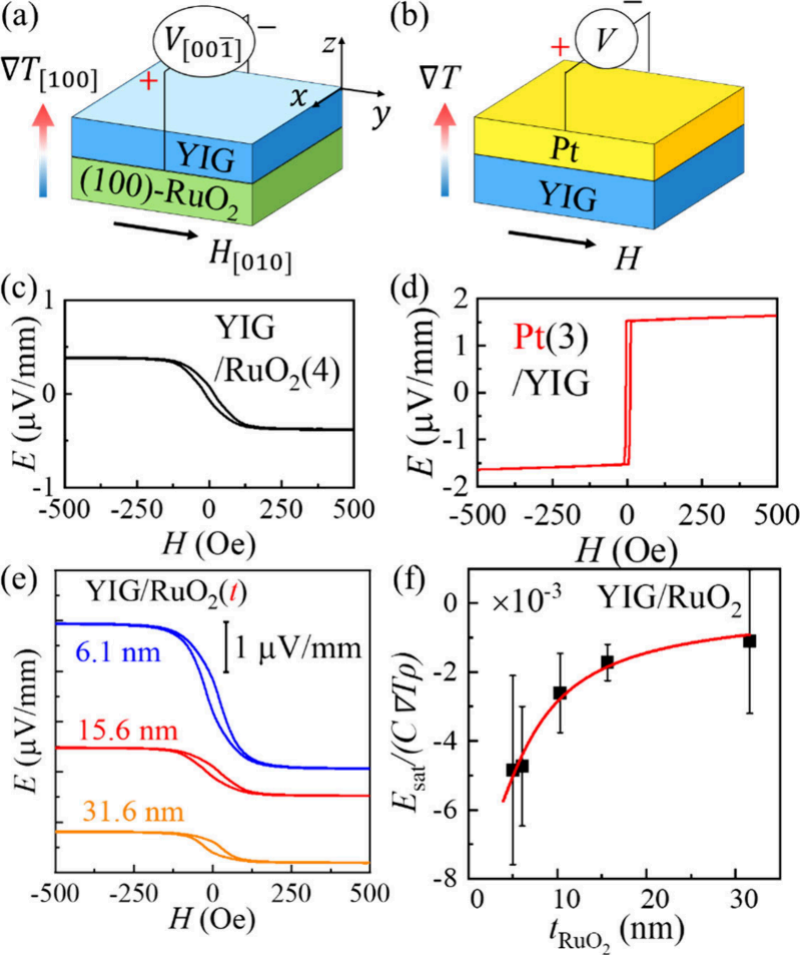
Schematic illustrations of the spin Seebeck measurements for the (a) YIG/RuO_2_/TiO_2_ and (b) Pt/YIG/GGG samples. The spin current injection direction follows the temperature gradient and thus is the same for (a) and (b), regardless of YIG layer sequence [19]. The spin Seebeck voltages are opposite for the (c) 3.9 nm-thick RuO_2_ and (d) 3 nm-thick Pt. Thickness-dependent (e) spin Seebeck electromotive force and (f) normalized voltage plot for RuO_2_.

Here, we provide a few possibilities that may contribute to the opposite sign for YIG/RuO_2_ and Py/RuO_2_. (1) The sizable and positive spin Hall effect in Py.^24,25^ This could result in an overall positive θ_SH_ for the Py/RuO_2_ heterostructure. (2) The metallic Ru state at the Py/RuO_2_ interface, as observed by our HAXPES measurement (see Supporting Information S12), which may significantly modify the spin-to-charge conversion at the interface. (3) Nontrivial Rashba states at the RuO_2_ surface,^8^ which may be preserved or vanish in proximity to YIG or Py. To conclusively identify the origin of the sign change, a careful and systematic analysis is necessary and awaits further theoretical and spectroscopic insights.

To quantify ${\theta_{\mathrm{SH}}}_{jk}^{i}$ , we perform thickness-dependent ISHE measurements on (100)-oriented RuO_2_, as shown in Figure 5e. Thicker films show smaller voltages due to spin diffusion. We fit the results in Figure 5f using Equation S11 in Supporting Information S13, and obtain a ${\theta_{SH}}_{bc}^{a}=-\left(4.0\pm 0.8\right)\%$ and *λ*_sd_ = 1.9 ± 0.5 nm. With the estimated resistivity for the bulk RuO_2_ as 157 μΩcm (see Supporting Information S14), we obtain the anisotropic spin Hall angles and conductivities for RuO_2_: ${\theta_{SH}}_{bc}^{a}\approx -\left(4.0\pm 0.8\right)\%$ , ${\theta_{SH}}_{ca}^{b}\approx -\left(0.3\pm 0.06\right)\%$ , ${\theta_{SH}}_{ab}^{c}\approx -\left(1.2\pm 0.2\right)\%$ , σ_*bc*_^*a*^ ≈ −250 ± 51 S cm^–1^, σ_*ca*_^*b*^ ≈ −19 ± 3 S cm^–1^, and σ_*ab*_^*c*^ ≈ −75 ± 15 S cm^–1^. These values are summarized in Table 1.

In conclusion, we systematically investigated the anisotropic spin-to-charge conversion in epitaxial RuO_2_ thin films across different crystal orientations and fabrication methods at room temperature. Most significantly, we show the absence of transport altermagnetic spin-splitting behavior in all of the RuO_2_ films we studied. Instead, we observe a robust anisotropic spin Hall effect with the spin Hall angle tensor components ${\theta_{SH}}_{bc}^{a}\approx -\left(4.0\pm 0.8\right)\%$ , ${\theta_{SH}}_{ca}^{b}\approx -\left(0.3\pm 0.06\right)\%$ , ${\theta_{SH}}_{ab}^{c}\approx -\left(1.2\pm 0.2\right)\%$ , directly obtained from our experiment. We also show that an unconventional *z*-spin accumulation is expected for the low-symmetry (101)-plane, which is intrinsic to RuO_2_ in the absence of magnetic order. Furthermore, we reveal a *negative* spin Hall angle for RuO_2_ when it is in contact with YIG, which changes to a positive sign when it is next to Py. Our study provides critical insights into the recent arguments regarding RuO_2_, and advances the understanding of spin-to-charge conversion in emerging altermagnetic materials with low crystal symmetries in general.

## Supplementary Material



## Notes

Sample characterizations using VSM, XRD, and TEM, discussions on the spin Hall conductivity tensors based on crystal symmetry, discussions on the magnetic ground state using magnetic field annealing treatment and XMCD, additional verifications of the experimental results via spin pumping techniques, and investigation of the interfacial Ru electronic states through HAXPES (PDF)
