# Supplementary material for: Absence of Transport Altermagnetic Spin-Splitting Effect in RuO2
Source: Nano Lett. 2026 Jan 29;26(7):2548–54. doi: 10.1021/acs.nanolett.5c05787 (PMC12947726; doi:10.1021/acs.nanolett.5c05787)
Supplement: Supplementary file 1 [file nl5c05787_si_001.pdf]

Supporting Information

Absence of transport altermagnetic spin-splitting effect in RuO_2

Yu-Chun Wang[1,2], Zhe-Yu Shen[1], Chia-Hsi Lin[1], Wei-Chih Hsu[1], You-Sheng Chen[2], Yi-Ying Chin[3], Akhilesh Kr. Singh[4], Wei-Li Lee[4], Chien-Te Chen[5], Ssu-Yen Huang[1,6&], and Danru Qu[2,6]*

[1] *Department of Physics, National Taiwan University, Taipei 10617, Taiwan*

[2] *Center for Condensed Matter Sciences, National Taiwan University, Taipei 10617, Taiwan*

[3] *Department of Physics, National Chung Cheng University, Chia-Yi 621301, Taiwan*

[4] *Institute of Physics, Academia Sinica, Taipei, 115201, Taiwan*

[5] *National Synchrotron Radiation Research Center, Hsinchu 300092, Taiwan*

[6] *Center of Atomic Initiatives for New Materials, National Taiwan University, Taipei 10617, Taiwan*

[&]syhuang@phys.ntu.edu.tw (S.Y.H.); [*]danru@ntu.edu.tw (D.Q.)

S1. Summary of crystal symmetry and the number of independent spin Hall conductivity components for altermagnetic materials.

In **Table S1**, we summarize the crystal space group (SG) number and the corresponding number of independent spin Hall conductivity (SHC) [S1] components for altermagnets [S2] exhibiting planar or bulk *d*-wave, *g*-wave, and *i*-wave spin-dependent Fermi surface symmetry. According to the symmetry analysis [S1], when the SG number is below 200, at least two independent SHC components are allowed, which we refer to as low crystal symmetry. Therefore, except for the bulk *i*-wave case, the other types of altermagnetic materials exhibit at least three independent SHC components, suggesting the importance of investigating anisotropic and unconventional spin Hall effects in these materials.

Spin moment locking	Laue group	# of SG	# of independent SHC	Candidates
Planar *d*-wave	*mmm*	16-74	6	La_2CuO_4,$FeSb_2$
	4/m	75-88	7	KRu_4O_8
	4/mmm	89-142	3	RuO_2, MnO_2, MnF_2
Planar *g*-wave				$KMnF_3$
Planar *i*-wave	*6/mmm*	177-194	3	
Bulk *d*-wave	*2/m*	3-15	13	CuF_2
Bulk *g*-wave	$\bar{3}/m$	149-167	4	CoF_3, FeF_3, Fe_2O_3
	6/m	168-176	7	
	6/mmm	177-194	3	CrSb, MnTe, VNb_3S_6
Bulk *i*-wave	$m\bar{3}m$	207-230	1	

Table S1. Laue group, space group number, number of independent spin Hall conductivity tensor components, and representative material candidates for altermagnets[S2], categorized by the symmetry of their spin-dependent Fermi surfaces.

S2. Magnetization measurements for YIG/RuO_2/TiO_2.

We measure the magnetization of the YIG/RuO_2/TiO_2 samples with various RuO_2 orientations and fabrication methods. We apply the magnetic field in the film plane in the *x* and *y* directions, which are parallel and perpendicular to the *c*-axis or its in-plane projections, respectively. The saturation magnetization for the 49 nm-, 53 nm-, and 50 nm-thick YIG films

grown on sputtered (110)-, (101)-, and MBE-grown (101)-oriented RuO_2 films are 1215 G, 1533 G, and 1470 G, respectively, as shown in **Figure S1a, b, and c**.

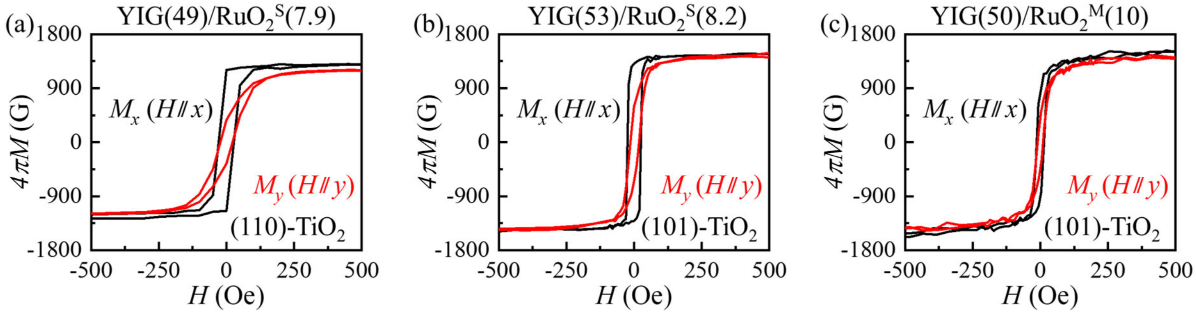

Fiigure S1. Magnetization measurements for a) YIG/RuO_2^S/$TiO_2^{(110)}$, b) YIG/RuO_2^S/$TiO_2^{(101)}$, and c) YIG/RuO_2^M/$TiO_2^{(101)}$. The *x*-axis is aligned parallel to the *c*-axis or its in-plane projections.

S3. *X*-ray diffraction spectroscopy and transmission electron microscopy for our RuO_2 films

We confirm the epitaxial relationship between the RuO_2 layer and the TiO_2 substrate using both *X*-ray diffraction spectroscopy (XRD) and transmission electron microscopy (TEM). For the 100 nm-thick RuO_2 films deposited on the (100)-, (110)-, and (101)-oriented TiO_2 substrates, single crystalline peaks are presented over a broad range of the XRD 2θ scan, as shown **in Figures S2a, c, and e**. Further XRD ϕ-scan in the insets of Figures S2a, c, and e present two peaks from the RuO_2 {110}, {200}, and {111} families, which match nicely with the corresponding peaks of the substrates, revealing the epitaxial relationship. For the thinner 10 nm-thick RuO_2 films capped with YIG layers, TEM images disclose sharp interfaces between the YIG and RuO_2 layers, and confirm the survival of the epitaxial relationship between RuO_2 and TiO_2 after the thermal treatment of the YIG layer, as shown in Figures S2b, d, and f. The fast Fourier transform (FFT) in

the insets, which shows the same patterns for the films and substrates, further confirms the epitaxial growth of RuO_2.

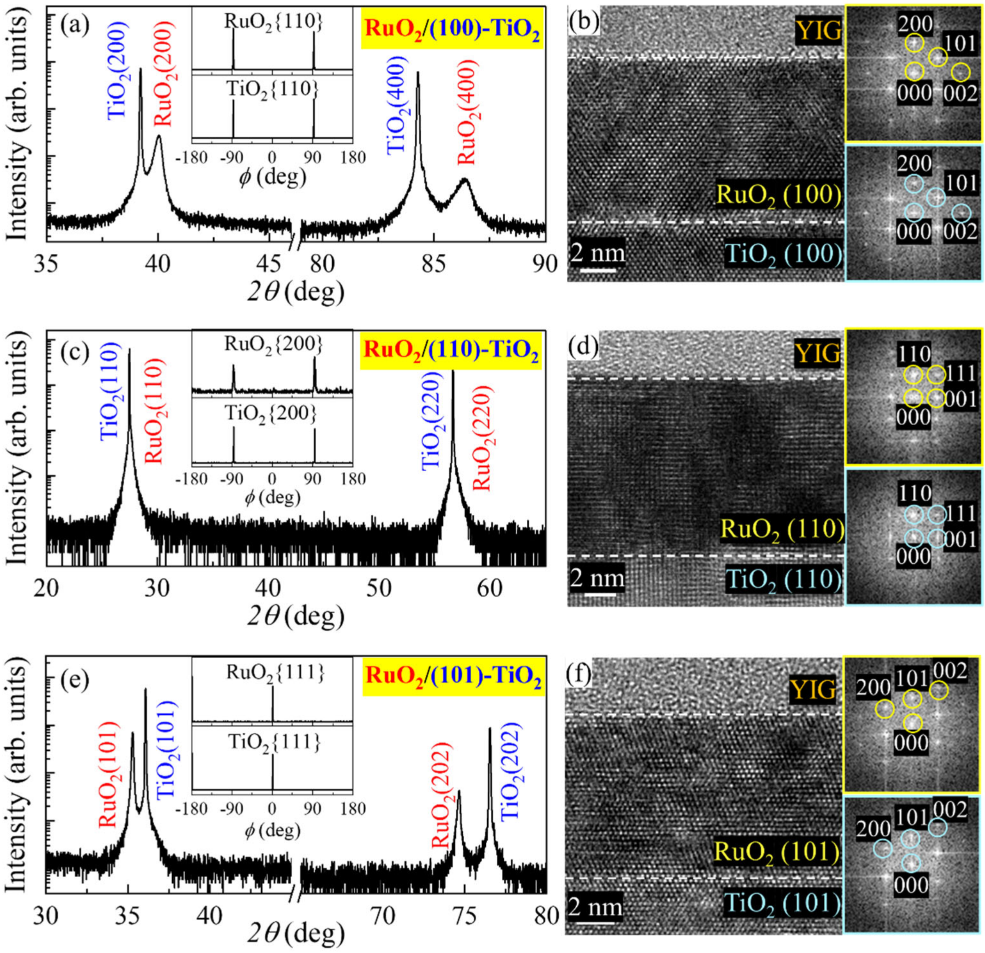

Figure S2. 2θ-scan X-ray diffraction (XRD) of the 100-nm-thick RuO_2 grown on the a) (100)-oriented, c) (110)-oriented, and e) (101)-oriented TiO_2 substrates, respectively. XRD ϕ-scan of {110}-planes of (100)-oriented RuO_2 and TiO_2, {200}-planes of (110)-oriented RuO_2 and TiO_2, and {111}-planes of (101)-oriented RuO_2 and TiO_2 are shown in the insets of a), c), and e), respectively. The cross-sectional TEM images of the b) YIG/RuO_2/$TiO_2^{(100)}$, d) YIG/RuO_2/$TiO_2^{(110)}$, and f) YIG/RuO_2/$TiO_2^{(101)}$, exhibiting a structural sharpness across the interfaces. Insets: FFT of the respective RuO_2 films (yellow-framed) and TiO_2 substrates (blue-framed).

S4. YIG grown on GGG substrate.

The gadolinium gallium garnet (GGG, a=12.382 Å) substrate has a small lattice mismatch compared with YIG (a=12.376 Å). We choose the (111)-oriented GGG substrate, denoted as $GGG^{(111)}$, to grow the single-crystalline YIG film via radio frequency (RF) magnetron sputtering, and deposit a 3 nm-thick Pt layer on top to detect the spin current. The crystal structures and orientations of YIG are determined by XRD, as shown in **Figure S3a**, which shows (222) and (444) single-crystalline peaks over a broad range of 2θ. Magnetic hysteresis loops of the films are plotted in Figure S3b. The single-crystal YIG shows a sizable magnetization of 1800 G.

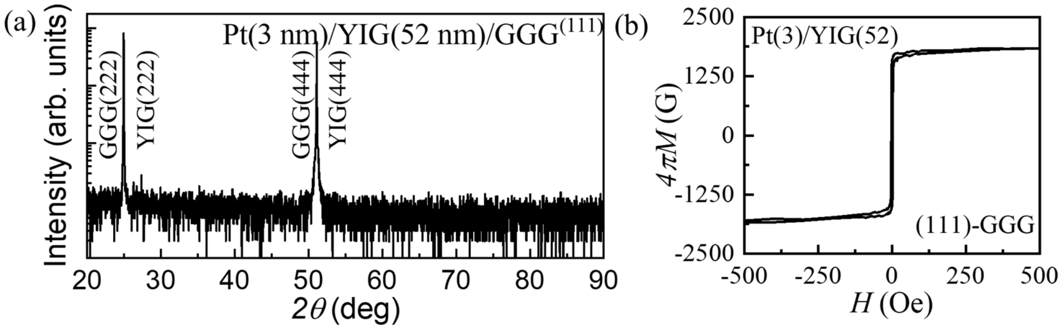

Figure S3. (a) XRD spectra and (b) magnetization measurement of Pt (3 nm)/YIG (52 nm)/$GGG^{(111)}$.

S5. Anisotropic spin Hall conductivity tensors.

We use a third-order tensor σ_{jk}^{i}, in which a spin current flowing along j with spin index i induces an electric field along k, to represent the spin Hall conductivity (SHC) element. Here, i, j, and k can correspond to any Cartesian coordinate x, y, or z; therefore, the SHC tensor has 27 elements. However, according to,[S1] strict symmetry constraints significantly reduce the number of independent elements in the spin Hall conductivity tensor in high-symmetry materials. The

conventional spin Hall effect (SHE) of rutile-structured RuO_2 (space group 136) has only three nonzero, independent elements $\sigma_{yz}^{x} = \sigma_{bc}^{a}$, $\sigma_{zx}^{y} = \sigma_{ca}^{b}$ and $\sigma_{xy}^{z} = \sigma_{ab}^{c}$. To calculate the spin Seebeck electric field measured along different crystal orientations, we project the SHC onto the (100)-, (110)-, and (101)-oriented RuO_2 planes using the transformation matrix D. The tensor operation is as follows:

$$\sigma'^{i}_{jk} = \sum_{lmn} D_{il} D_{jm} D_{kn} \sigma_{mn}^{l} \tag{S1}$$

Where σ'^{i}_{jk} denotes the SHC tensor element after coordinate transformation, while σ_{mn}^{l} represents the tensor element for RuO_2 (001). As shown in **Table S2**, we list the SHC tensors of RuO_2 (100), RuO_2 (110), and RuO_2 (101). We also list in **Table S3** the ASSE tensor in different RuO_2 orientations.

SHE, RuO_2 (100), $x \parallel [00\bar{1}]$, $y \parallel [010]$	
σ^x	$\begin{bmatrix} 0 & 0 & 0 \\ 0 & 0 & \sigma_{ab}^{c} \\ 0 & -\sigma_{ab}^{c} & 0 \end{bmatrix}$
σ^y	$\begin{bmatrix} 0 & 0 & -\sigma_{ca}^{b} \\ 0 & 0 & 0 \\ \sigma_{bc}^{a} & 0 & 0 \end{bmatrix}$
σ^z	$\begin{bmatrix} 0 & \sigma_{ca}^{b} & 0 \\ -\sigma_{bc}^{a} & 0 & 0 \\ 0 & 0 & 0 \end{bmatrix}$
SHE, RuO_2 (110), $x \parallel [00\bar{1}]$, $y \parallel [\bar{1}10]$	
σ^x	$\begin{bmatrix} 0 & 0 & 0 \\ 0 & 0 & \sigma_{ab}^{c} \\ 0 & -\sigma_{ab}^{c} & 0 \end{bmatrix}$

σ^y	$\begin{bmatrix} 0 & 0 & -\sigma_{ca}^{b} \\ 0 & 0 & 0 \\ \sigma_{bc}^{a} & 0 & 0 \end{bmatrix}$
σ^z	$\begin{bmatrix} 0 & \sigma_{ca}^{b} & 0 \\ -\sigma_{bc}^{a} & 0 & 0 \\ 0 & 0 & 0 \end{bmatrix}$
SHE, RuO_2 (101), $x \parallel [10\bar{1}]$, $y \parallel [010]$	
σ^x	$\begin{bmatrix} 0 & (\sigma_{ca}^{b} - \sigma_{ab}^{c}) \, sin\, \theta_c \, cos\, \theta_c & 0 \\ (\sigma_{ab}^{c} - \sigma_{bc}^{a}) \, sin\, \theta_c \, cos\, \theta_c & 0 & \sigma_{ab}^{c} \, sin^2\, \theta_c + \sigma_{bc}^{a} \, cos^2\, \theta_c \\ 0 & -\sigma_{ab}^{c} \, sin^2\, \theta_c - \sigma_{ca}^{b} \, cos^2\, \theta_c & 0 \end{bmatrix}$
σ^y	$\begin{bmatrix} (\sigma_{bc}^{a} - \sigma_{ca}^{b}) \sin \theta_c \cos \theta_c & 0 & -\sigma_{ca}^{b} \sin^2 \theta_c - \sigma_{bc}^{a} \cos^2 \theta_c \\ 0 & 0 & 0 \\ \sigma_{ca}^{b} \cos^2 \theta_c + \sigma_{bc}^{a} \sin^2 \theta_c & 0 & (\sigma_{ca}^{b} - \sigma_{bc}^{a}) \sin \theta_c \cos \theta_c \end{bmatrix}$
σ^z	$\begin{bmatrix} 0 & \sigma_{ab}^{c} \, cos^2\, \theta_c + \sigma_{ca}^{b} \, sin^2\, \theta_c & 0 \\ -\sigma_{ab}^{c} \, cos^2\, \theta_c - \sigma_{bc}^{a} \, sin^2\, \theta_c & 0 & (\sigma_{bc}^{a} - \sigma_{ab}^{c}) \, sin\, \theta_c \, cos\, \theta_c \\ 0 & (\sigma_{ab}^{c} - \sigma_{ca}^{b}) \, sin\, \theta_c \, cos\, \theta_c & 0 \end{bmatrix}$

Table S2. SHE induced SHC tensors for RuO_2 (100), where the *x*- and *y*-axes lie along the $[00\bar{1}]$ and [010] directions, respectively. For RuO_2 (110), the *x*- and *y*-axes lie along the $[00\bar{1}]$ and $[\bar{1}10]$ directions, respectively. For RuO_2 (101), the *x*- and *y*-axes lie along the $[10\bar{1}]$ and [010] directions, respectively. θ_c = 34.56° denotes the angle between the (001) and (101) planes.

ASSE, RuO_2 (100), $x \parallel [00\bar{1}]$, $y \parallel [010]$	
σ^x	$\begin{bmatrix} 0 & 0 & 0 \\ 0 & 0 & -\sigma_{ASSE} \\ 0 & -\sigma_{ASSE} & 0 \end{bmatrix}$
σ^y	$\begin{bmatrix} 0 & 0 & 0 \\ 0 & 0 & 0 \\ 0 & 0 & 0 \end{bmatrix}$

σ^z	$\begin{bmatrix} 0 & 0 & 0 \\ 0 & 0 & 0 \\ 0 & 0 & 0 \end{bmatrix}$
ASSE, RuO_2 (110), $x \parallel [00\bar{1}]$, $y \parallel [\bar{1}10]$	
σ^x	$\begin{bmatrix} 0 & 0 & 0 \\ 0 & \sigma_{ASSE} & 0 \\ 0 & 0 & -\sigma_{ASSE} \end{bmatrix}$
σ^y	$\begin{bmatrix} 0 & 0 & 0 \\ 0 & 0 & 0 \\ 0 & 0 & 0 \end{bmatrix}$
σ^z	$\begin{bmatrix} 0 & 0 & 0 \\ 0 & 0 & 0 \\ 0 & 0 & 0 \end{bmatrix}$
ASSE, RuO_2 (101), $x \parallel [10\bar{1}]$, $y \parallel [010]$	
σ^x	$\begin{bmatrix} 0 & -\sigma_{ASSE} \sin\theta_N \cos\theta_N & 0 \\ -\sigma_{ASSE} \sin\theta_N \cos\theta_N & 0 & -\sigma_{ASSE} \sin^2\theta_N \\ 0 & -\sigma_{ASSE} \sin^2\theta_N & 0 \end{bmatrix}$
σ^y	$\begin{bmatrix} 0 & 0 & 0 \\ 0 & 0 & 0 \\ 0 & 0 & 0 \end{bmatrix}$
σ^z	$\begin{bmatrix} 0 & \sigma_{ASSE} \cos^2\theta_N & 0 \\ \sigma_{ASSE} \cos^2\theta_N & 0 & \sigma_{ASSE} \sin\theta_N \cos\theta_N \\ 0 & \sigma_{ASSE} \sin\theta_c \cos\theta_N & 0 \end{bmatrix}$

Table S3. ASSE-induced conductivity tensors for RuO_2 (100), where the *x*- and *y*-axes lie along the $[00\bar{1}]$ and [010] directions, respectively. For RuO_2 (110), the *x*- and *y*-axes lie along the $[00\bar{1}]$ and $[\bar{1}10]$ directions, respectively. For RuO_2 (101), the *x*- and *y*-axes lie along the $[10\bar{1}]$ and [010] directions, respectively. θ_N denotes the angle between the Néel vector and the normal to the RuO_2 (101) surface.

For the ASSE, only one nonzero, independent element $\sigma_{xy}^{z} = \sigma_{yx}^{z} = \sigma_{ASSE}$ exists in RuO_2 (001). We perform the same tensor operation on ASSE-induced SHC tensors in different crystal orientations of RuO_2, and the results are shown in Table S3.

We measure the electric field E_x (E_y) along the $+x$ ($-y$) direction, with a vertical temperature gradient along the $+z$ direction and a magnetic field applied along the $+y$ ($+x$) direction. We use the following equations to obtain the voltage ratios for E_y and E_x:

$$\frac{E_y}{E_x} = \frac{-\sigma_{zy}^{x}}{\sigma_{zx}^{y}} \tag{S2}$$

Therefore, the E_y/E_x ratio of RuO_2 (100) is expressed as

$$\frac{E_y}{E_x} = \frac{\sigma_{ab}^{c} + \sigma_{ASSE}}{\sigma_{bc}^{a}} \tag{S3}$$

The E_y/E_x ratio of RuO_2 (110) is expressed as

$$\frac{E_y}{E_x} = \frac{\sigma_{ab}^{c}}{\sigma_{bc}^{a}} \tag{S4}$$

The E_y/E_x ratio of RuO_2 (101) is expressed as

$$\frac{E_y}{E_x} = \frac{\sigma_{ab}^{c}\, sin^2\,\theta_c + \sigma_{ca}^{b}\, cos^2\,\theta_c + \sigma_{ASSE}\, sin^2\,\theta_N}{\sigma_{ca}^{b}\cos^2\theta_c + \sigma_{bc}^{a}\sin^2\theta_c} \tag{S5}$$

S6. Unconventional spin Hall effect.

For low-crystal-symmetry planes such as the (101)-plane, besides the contribution from σ_{zx}^{y} and σ_{zy}^{x} to E_x and E_y, additional nonzero and unconventional contributions also arise from σ_{zx}^{x} and σ_{zy}^{y}. The magnetic field angular dependence of the E_x and E_y is thus described by the following expressions:

$$\begin{aligned} E_x(\phi) &\propto +\sigma_{zx}^{x}\cos\phi + \sigma_{zx}^{y}\sin\phi \\ E_y(\phi) &\propto -\sigma_{zy}^{x}\cos\phi - \sigma_{zy}^{y}\sin\phi \end{aligned} \qquad \text{(S6)}$$

To show that these unconventional spin Hall components exist for the (101)-plane, we cut a square-shaped (101)-RuO_2 sample with the *x*- and *y*-axis rotated 45° counterclockwise from the $[10\bar{1}]$ and [010] directions, respectively, denoted as the 45°-cut sample. The SHC in the 45°-cut sample is shown in **Table S4**.

SHE, RuO_2 (101), *x* and *y* rotated counterclockwise an angle φ with respect to $[10\bar{1}]$ and [010]	
σ^x	$\begin{bmatrix} 0 & (\sigma_{ca}^{b}-\sigma_{ab}^{c})\sin\theta_c\cos\theta_c\cos\varphi & (\sigma_{ab}^{c}-\sigma_{ca}^{b})\sin^2\theta_c\sin\varphi\cos\varphi \\ (\sigma_{ab}^{c}-\sigma_{bc}^{a})\sin\theta_c\cos\theta_c\cos\varphi & (\sigma_{bc}^{a}-\sigma_{ca}^{b})\sin\theta_c\cos\theta_c\sin\varphi & \sigma_{ab}^{c}\sin^2\theta_c\cos^2\varphi+\sigma_{ca}^{b}\sin^2\theta_c\sin^2\varphi+\sigma_{bc}^{a}\cos^2\theta_c \\ (\sigma_{bc}^{a}-\sigma_{ab}^{c})\sin^2\theta_c\sin\varphi\cos\varphi & -\sigma_{ab}^{c}\sin^2\theta_c\cos^2\varphi-\sigma_{ca}^{b}\cos^2\theta_c-\sigma_{bc}^{a}\sin^2\theta_c\sin^2\varphi & (\sigma_{ca}^{b}-\sigma_{bc}^{a})\sin\theta_c\cos\theta_c\sin\phi \end{bmatrix}$
σ^y	$\begin{bmatrix} (\sigma_{bc}^{a}-\sigma_{ca}^{b})\sin\theta_c\cos\theta_c\cos\varphi & (\sigma_{ab}^{c}-\sigma_{bc}^{a})\sin\theta_c\cos\theta_c\sin\varphi & -\sigma_{ab}^{c}\sin^2\theta_c\, sin^2\varphi-\sigma_{ca}^{b}\sin^2\theta_c\cos^2\varphi-\sigma_{bc}^{a}\cos^2\theta_c \\ (\sigma_{ca}^{b}-\sigma_{ab}^{c})\sin\theta_c\cos\theta_c\sin\varphi & 0 & (\sigma_{ca}^{b}-\sigma_{ab}^{c})\sin^2\theta_c\sin\varphi\cos\varphi \\ \sigma_{ab}^{c}\sin^2\theta_c\sin^2\varphi+\sigma_{ca}^{b}\cos^2\theta_c+\sigma_{bc}^{a}\sin^2\theta_c\cos^2\varphi & (\sigma_{ab}^{c}-\sigma_{bc}^{a})\sin^2\theta_c\sin\varphi\cos\varphi & (\sigma_{ca}^{b}-\sigma_{bc}^{a})\sin\theta_c\cos\theta_c\cos\varphi \end{bmatrix}$
σ^z	$\begin{bmatrix} (\sigma_{ca}^{b}-\sigma_{bc}^{a})\sin^2\theta_c\sin\varphi\cos\varphi & \sigma_{ab}^{c}\cos^2\theta_c+\sigma_{ca}^{b}\sin^2\theta_c\cos^2\varphi+\sigma_{bc}^{a}\sin^2\theta_c\sin^2\varphi & (\sigma_{ab}^{c}-\sigma_{bc}^{a})\sin\theta_c\cos\theta_c\sin\varphi \\ -\sigma_{ab}^{c}\cos^2\theta_c-\sigma_{ca}^{b}\sin^2\theta_c\sin^2\varphi-\sigma_{bc}^{a}\sin^2\theta_c\cos^2\varphi & (\sigma_{bc}^{a}-\sigma_{ca}^{b})\sin^2\theta_c\sin\varphi\cos\varphi & (\sigma_{ab}^{c}-\sigma_{bc}^{a})\sin\theta_c\cos\theta_c\cos\varphi \\ (\sigma_{ca}^{b}-\sigma_{ab}^{c})\sin\theta_c\cos\theta_c\sin\varphi & (\sigma_{ca}^{b}-\sigma_{ab}^{c})\sin\theta_c\cos\theta_c\cos\varphi & 0 \end{bmatrix}$

Table S4. SHE induced SHC tensors for RuO_2 (101). θ_c = 34.56° denotes the angle between the (001) and (101) plane. $\varphi = 45°$ denotes the angle between the *x* and [010].

For the original cut sample, with y-axis aligned with the [010]-direction, E_x and E_y is proportional to

$$E_x(\phi) \propto (0.68\sigma_{ca}^{b} + 0.32\sigma_{bc}^{a})\sin\phi$$
$$E_y(\phi) \propto (0.32\sigma_{ab}^{c} + 0.68\sigma_{ca}^{b})\cos\phi \quad \text{(S7)}$$

Using our experimental data with $\sigma_{ca}^{b}/\sigma_{bc}^{a} \approx 8$ % and $\sigma_{ab}^{c}/\sigma_{bc}^{a} \approx 30$ %, we obtain:

$$E_x(\phi)/(-\sigma_{bc}^{a}) \propto -0.376\sin\phi$$
$$E_y(\phi)/(-\sigma_{bc}^{a}) \propto -0.151\cos\phi \quad \text{(S8)}$$

The simulated magnetic field angular dependence of E_x and E_y is plotted in **Figure S4c**.

For the 45°-cut sample, with the x- and y-axis rotated counterclockwise 45° off the $[10\bar{1}]$ and [010] directions, E_x and E_y is proportional to

$$E_x(\phi) \propto +0.16(\sigma_{bc}^{a} - \sigma_{ab}^{c})\cos\phi + (0.16\sigma_{ab}^{c} + 0.68\sigma_{ca}^{b} + 0.16\sigma_{bc}^{a})\sin\phi$$
$$E_y(\phi) \propto +(0.16\sigma_{ab}^{c} + 0.68\sigma_{ca}^{b} + 0.16\sigma_{bc}^{a})\cos\phi + 0.16(\sigma_{bc}^{a} - \sigma_{ab}^{c})\sin\phi \quad \text{(S9)}$$

Using our experimental data with $\sigma_{ca}^{b}/\sigma_{bc}^{a} \approx 8$ % and $\sigma_{ab}^{c}/\sigma_{bc}^{a} \approx 30$ %, we obtain:

$$E_x(\phi)/(-\sigma_{bc}^{a}) \propto -0.113\cos\phi - 0.263\sin\phi$$
$$E_y(\phi)/(-\sigma_{bc}^{a}) \propto -0.263\cos\phi - 0.113\sin\phi \quad \text{(S10)}$$

In contrast to the regular cut, for the 45°-cut sample, the maximum value for E_x and E_y are comparable, while the minimum value of E_x and E_y are located respectively at ϕ = 67° and 23°. The simulated magnetic field angular dependence of E_x and E_y is plotted in Figure S4d. Both simulations in Figure S4 show excellent agreement, in both magnitude and phase, with the experimental data shown in Figure 3 in the main text, further confirming the robustness and consistency of the anisotropic spin Hall effect in RuO_2.

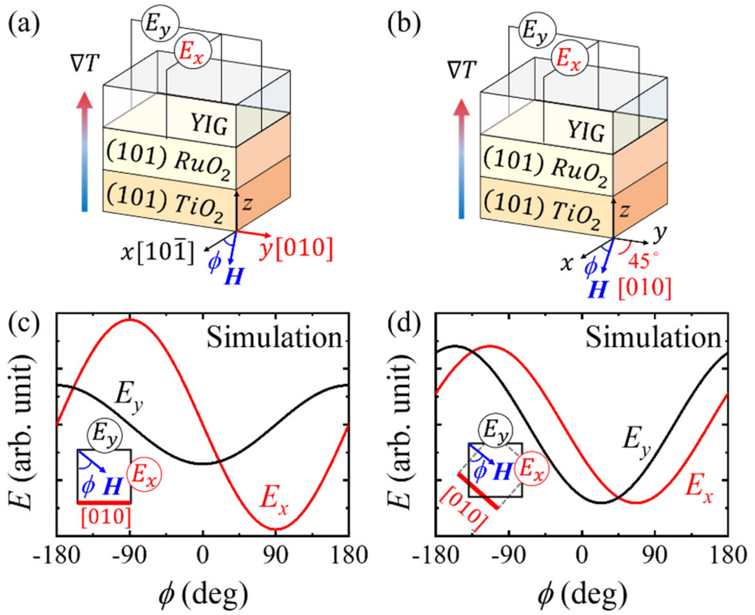

Figure S4. Simulated magnetic field angular dependent E_x and E_y for the (101)-oriented YIG/RuO_2/TiO_2 samples with a)c) regular cut and b)d) 45°-cut samples. The bonding wires directly contact the RuO_2 layer for voltage measurements. The angle ϕ denotes the orientation of the external magnetic field *H* relative to the *x*-axis.

S7. Robust anisotropic voltage ratio before and after magnetic field annealing.

In the magnetic field annealing process, the sample is placed under a nitrogen atmosphere. The temperature is increased from room temperature up to 473 K, well above the expected Néel temperature of RuO_2, which is about 400 K, and maintained for 2 hours. A magnetic field (H_{FA}) of 1.8 T is applied sequentially along the [010]- (red), [00$\bar{1}$]- (blue), [001]- (cyan), and [100]- (purple) directions to the RuO_2 crystal during four different annealing processes as illustrated in Figure S5a. As shown in Figure S5b and c, a consistent E_y/E_x ratio of about 30 % is observed before and after the magnetic field annealing processes. Since the annealing temperature is well above the reported Néel temperature (about 400 K) of RuO_2,[S3] a magnetic field of 1.8 T should

deterministically set the Néel order of RuO_2, which is in proximity with the magnetic insulator YIG. However, the nearly unaltered SSE signal supports the absence of an altermagnetic spin-splitting contribution to the observed SSE signals. It also provides essential evidence for the absence of magnetic order in RuO_2.

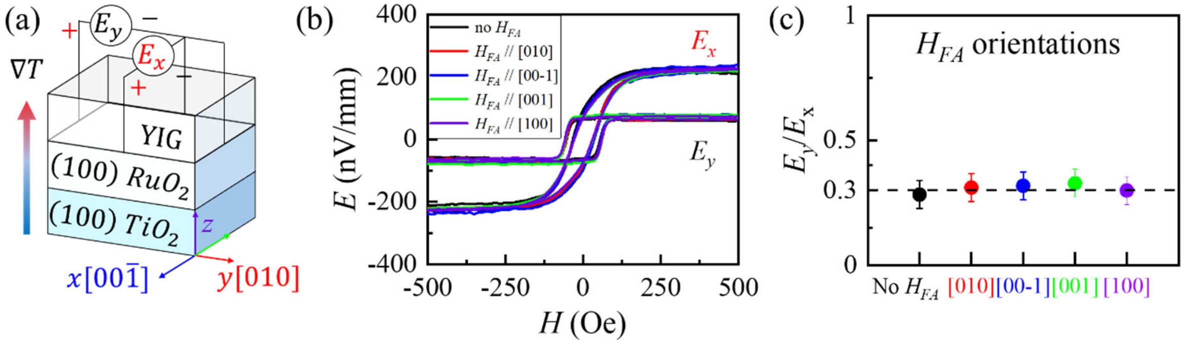

Figure S5. a) Schematic illustrations of the experiment setup for (100)-oriented YIG (45)/RuO_2 (10)/TiO_2 sample. The bonding wires directly contact the RuO_2 layer for voltage measurements. Different magnetic field annealing H_{FA} directions are distinguished by different colors in the illustrations. b) The spin Seebeck voltages obtained before and after the annealing process. c) Summary of H_{FA} direction-dependent E_y/E_x ratios.

S8. The X-ray magnetic circular dichroism experiments

The X-ray magnetic circular dichroism (XMCD) experiments were conducted at TLS-BL11A of the National Synchrotron Radiation Research Center in Taiwan. The Ru-$M_{2,3}$ spectra were recorded at room temperature using the total electron yield mode, achieving an energy resolution of better than 0.25 eV. To calibrate the photon energy, a $SrTiO_3$ thin film was simultaneously measured in a separate chamber with an accuracy of better than 10 meV. The degree of X-ray circular polarization was approximately 75%. The XMCD spectra were derived

as the difference between the XAS spectra with the circular polarization vector aligned parallel and antiparallel to the external magnetic field of 1.0 T, oriented normal to the surface.

XMCD experiments were conducted to investigate the presence of ferromagnetic ordering in the 10 nm-thick RuO_2 deposited on the (100)-oriented TiO_2 substrate. The applied external magnetic field was 1.0 T, aligned parallel to the surface normal. All spectra were obtained using the total electron yield mode and were normalized to the intensity of the incident beam. The XMCD measurements at the Ru-$M_{2,3}$ edges, as shown in **Figure S6**, revealed no discernible XMCD signal. The absence of a detectable XMCD signal signifies either the lack of a net magnetic moment of Ru or the presence of a magnetic moment that is too weak or unfavorably oriented to generate a measurable signal under a 1.0 T magnetic field.

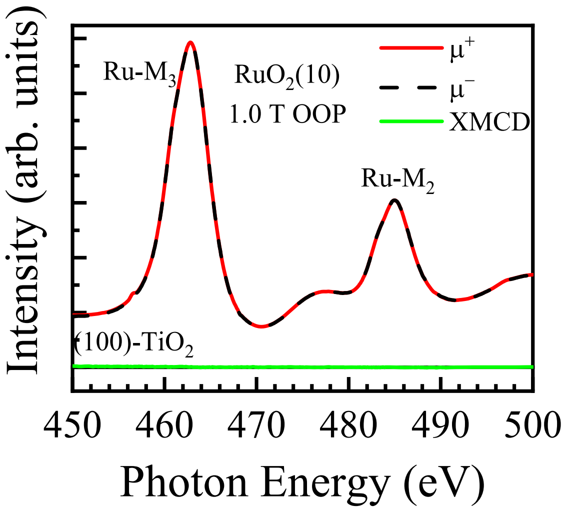

Figure S6. The XMCD measurements at the Ru-$M_{2,3}$ edges.

S9. Effect of the stacking order.

The stacking order does not affect the sign of the ISHE signal. The commonly used SSE geometry is illustrated in **Figure S7a**, where Pt is placed on top of YIG, a temperature gradient is applied along the *z*-axis, a magnetic field is applied along the *y*-axis, and the voltage is measured

along the x-axis. Next, we rotate the whole setup by 180° about the magnetic field axis, as shown in Figure S7b. This is equivalent to viewing the entire setup from an upside-down perspective. In this case, the sign of the signal is unchanged. Finally, we reverse both the direction of the temperature gradient and the wire connections of the voltmeter. This operation also retains the sign of the signal. As a result, the measurement configuration in Figure S7c is equivalent to that in Figure S7a, confirming that the ISHE sign is independent of the stacking order.

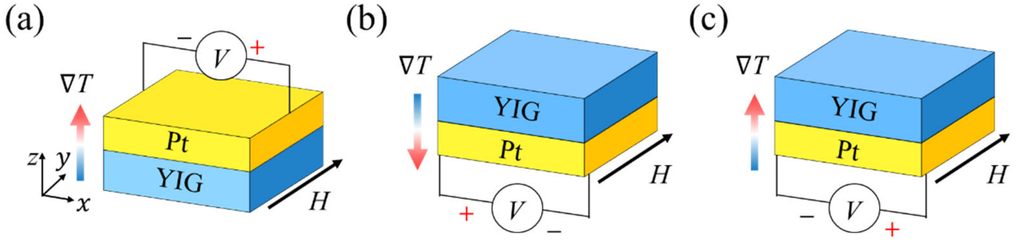

Figure S7. Illustration of the invariance of the ISHE signal with respect to the film stacking order. a) Conventional measurement configuration. b) Configuration view from an upside-down perspective. c) Simultaneous reversal of the temperature gradient and voltmeter connections preserves the sign of the detected voltage. As a result, configuration c) is equivalent to a).

S10. Summarization of literature reported spin Hall angles or spin-torque efficiency for RuO_2.

In **Table S5**, we summarize the spin Hall angles (θ_{SH}) or spin-torque efficiencies (ξ_{DL}) reported in the literature. Using a ferromagnetic metal layer, such as permalloy (Py), as a spin current source or detector, a positive θ_{SH} for RuO_2 has been obtained. We consider the positive θ_{SH} for Py and the interfacial Ru with reduced valence state to be responsible for the positive θ_{SH} or ξ_{DL} for Py/RuO_2.

RuO_2	θ_{SH} or ξ_{DL} (%)	FM	Method	Ref.
(100)	+4.6	Py	ST-FMR	[S4]
	+2.0	Py	ST-FMR	[S5]
(110)	+2.0	Py	ST-FMR	[S5]
	+4.4	Py	Spin pumping	[S6]
(101)	+1.0	Py	ST-FMR	[S4]
	+4.0	Py	ST-FMR	[S7]
	+3.5	Py	Spin pumping	[S6]
	+1.4	Co	Current switching	[S8]
(001)	+3.8	Py	ST-FMR	[S4]
	+5.4	Py	ST-FMR	[S7]

Table S5. Spin Hall angle for rutile RuO_2 in different crystal orientations, extracted from the literature using ferromagnetic metals.

S11. Spin pumping experiments on Py/Pt, Py/RuO_2, and YIG/RuO_2.

In our previous studies, by comparing methods such as spin pumping [S9] and spin-orbit torque magnetization switching measurements,[S10] we have demonstrated that thermal spin injection from YIG is an effective method for determining the sign of θ_{SH} across various materials, as YIG is an insulator and introduces minimal artifacts.

To further verify the spin Hall angle sign difference for RuO_2 when it is in contact with Py and YIG, we also perform the spin pumping experiments at room temperature using a TE_{102} cavity with a microwave frequency of 9.8 GHz. Three samples are used in the spin pumping experiments, Al(1 nm)/Py(15 nm)/Pt(10 nm) deposited onto a SiO_2/Si substrate, Al(1 nm)/Py(15 nm)/RuO_2(10

nm) deposited onto a (100)-oriented TiO_2 substrate, and YIG(50 nm)/RuO_2(10 nm) deposited onto a (100)-oriented TiO_2 substrate, as shown in **Figure S8 a, b, c**, respectively. A 1 nm-thick Al capping layer is used to prevent oxidation of the Py layer.

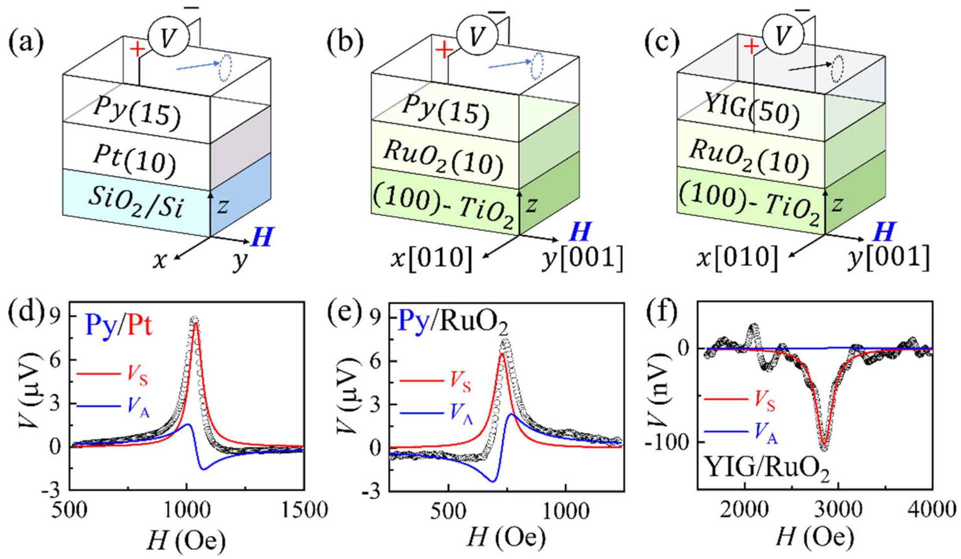

Figure S8. Schematic illustrations of the experiment setup for a) Py/Pt, b) Py/RuO_2, and c) YIG/RuO_2. The spin pumping voltage as a function of magnetic field for d) Py/Pt, e) Py/RuO_2, and f) YIG/RuO_2. The bonding wires directly contact the RuO_2 layer for voltage measurements. Solid and dashed lines are fits to symmetric Lorentzian V_S and antisymmetric Lorentzian V_A, respectively.

All samples follow the same FM/HM or FMI/HM stacking order to inject spin current along the - z direction, with spin polarization along the + y axis. The mixing voltage V is captured along the + x direction and decomposed into the symmetric (V_S) and asymmetric (V_A) voltages. The symmetric term V_S is induced by the spin-to-charge conversion (ISHE) in the HM, while the asymmetric term V_S is induced by the rectification effects, such as the anomalous Hall effect or anisotropic magnetoresistance, in the FM layer.

By focusing on V_S, we note that for both Py/Pt and Py/RuO_2, as shown in Figure S8d and e, a positive ISHE signal is observed under a microwave power of 68 mW and 11 mW, respectively. In

contrast, for the YIG/RuO_2 sample, measured under a microwave power of 54 mW, as shown in Figure S8f, a negative ISHE signal is observed. The significantly reduced voltage and increased noise level are caused by the reduced spin current generated from the polycrystalline YIG thin film layer.[S11] This negative ISHE voltage from the spin pumping measurements for YIG/RuO_2, compared with that in Py/RuO_2 and Py/Pt, is consistent with the SSE results, experimentally confirming the negative spin Hall angle for RuO_2.

S12. The hard X-ray photoelectron spectroscopy (HAXPES) experiments.

The hard X-ray photoelectron spectroscopy (HAXPES) experiments were conducted at the Max Planck-NSRRC end station at the BL12XU at SPring-8 in Hyogo, Japan. The photon beam was linearly polarized with the electric field vector in the plane of the storage ring (i.e., horizontal), and the photon energy was set at approximately 6.5 keV. The experimental setup included two MB Scientific A-1 HE analyzers, with the horizontally mounted analyzer being used. The Fermi edge of the gold film was measured to calibrate the photon energy and obtain the energy resolution. The overall energy resolution is estimated to be 0.27 eV by fitting the Fermi edge of gold.

As shown in **Figure S9a**, for the Ru-$3d_{5/2}$ HAXPES spectra, a main peak at 280.8 eV, marked by the black arrow, is observed corresponding to the Ru^{4+} state [S12-S15] for all three samples. However, for the Py/RuO_2 spectrum (red), an additional step is observed at around 280 eV, marked by the red arrow, indicating the lower valence state of Ru. The simulated spectra, as shown in Figure S9b for Py/RuO_2 and YIG/RuO_2, reveal that the step at 280 eV for Py/RuO_2 corresponds to the presence of a Ru metal state.[S12, S13, S15] All the RuO_2 films are sputtered onto the (100)-oriented TiO_2 substrate.

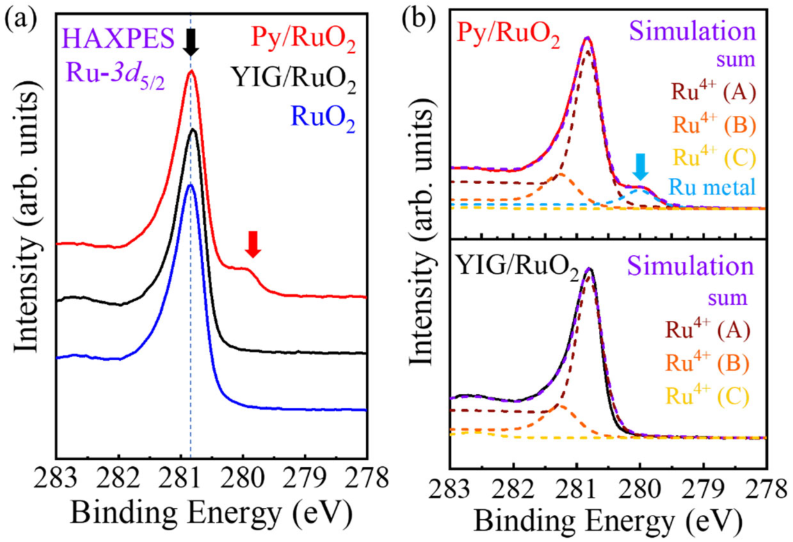

Figure S9. a) HAXPES spectra and b) simulations for Py/RuO_2 (red), YIG/RuO_2 (black), and RuO_2 (blue). All the RuO_2 films are sputtered onto the (100)-oriented TiO_2 substrate.

The photon energies and the ratio of integrated areas are listed in **Table S6**. According to the intensity ratios of Ru^{4+} and Ru metal features in $Ni_{80}Fe_{20}/RuO_2/TiO_2$, around 10% Ru is reduced to the Ru metal state.

$Ni_{80}Fe_{20}/RuO_2/TiO_2$

	Photon Energy (eV)	ratio
Ru metal	280.00	9.6%
Ru^{4+} (A)	280.82	70.6%
Ru^{4+} (B)	281.30	19.1%
Ru^{4+} (C)	282.60	0.7%

$Y_3Fe_5O_{12}/RuO_2/TiO_2$

	Photon Energy (eV)	ratio
Ru^{4+} (A)	280.80	77.7%
Ru^{4+} (B)	281.25	18.7%
Ru^{4+} (C)	282.60	3.6%

Table S6. The photon energies and the ratio of integrated areas of the fitted features attributed to Ru^{4+} and Ru metal.

S13. Estimation of the spin Hall angle θ_{SH} and spin diffusion length λ_{sd}.

To estimate the θ_{SH} and λ_{sd} for RuO_2, we use the following equation [S16]

$$E_{sat}(t) = C\nabla T \rho(t) \theta_{SH} \frac{\lambda_{sd}}{t} \tanh\left(\frac{t}{2\lambda_{sd}}\right) \quad \text{(S11)}$$

Here, E_{sat} represents the saturation SSE electric field, ∇T = 13 K mm^{-1} is the temperature gradient, estimated from an applied heat flux of $Q = 10^5$ W m^{-2} and the YIG thermal conductivity κ= 7.4 Wm^{-1}K^{-1}, ρ is the resistivity of RuO_2, and t denotes the thickness of the RuO_2 film. Parameter C, the spin current injection coefficient, encompasses the magnetic properties of YIG. By using the gyromagnetic ratio γ = 2.76×10^{10} s^{-1} T^{-1} and the spin mixing conductance $g_{eff}^{\uparrow\downarrow}$ =3.67×10^{18} m^{-2}, into the equation, we estimate C to be 4.3 A m^{-1} K^{-1}. Following Equation S11, we plot $E_{sat}/(C\nabla T\rho)$ to estimate θ_{SH} and λ_{sd} for RuO_2 at varying thicknesses, as shown in Figure 4 (f) in the main text. The fitting yields θ_{SH} = - (4.0 ± 0.8)% and λ_{sd} = 1.9 ± 0.5 nm.

S14. Estimation of the bulk RuO_2 resistivity.

Figure S10 illustrates the dependence of the ρ along the c-axis on the thickness of the RuO_2 layer. For thicker films, ρ remains nearly constant; however, as the film thickness decreases, ρ increases due to enhanced surface scattering effects. The red line is fitted using the Fuchs-Sondheimer model, and the fitted bulk resistivity for RuO_2 is 157 μΩ cm.

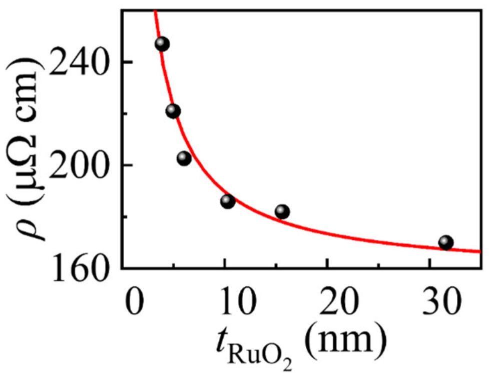

Figure S10. Thickness-dependent resistivity along the *c*-axis of RuO_2 thin films on TiO_2 (100). The solid red lines fit using the Fuchs-Sondheimer formalism.

References:

S1. A. Roy, *et al.*, Unconventional spin Hall effects in nonmagnetic solids, Phys. Rev. Mater. **6**, 045004 (2022).

S2. L. Šmejkal, *et al.*, Beyond Conventional Ferromagnetism and Antiferromagnetism: A Phase with Nonrelativistic Spin and Crystal Rotation Symmetry, Phys. Rev. X **12**, 031042 (2022).

S3. Z. Feng, *et al.*, An anomalous Hall effect in altermagnetic ruthenium dioxide, Nat. Electronics **5**, 735 (2022).

S4. S. Karube, *et al.*, Observation of Spin-Splitter Torque in Collinear Antiferromagnetic RuO_2, Phys. Rev. Lett. **129**, 137201 (2022).

S5. H. Bai, *et al.*, Observation of Spin Splitting Torque in a Collinear Antiferromagnet RuO_2, Phys. Rev. Lett. **128**, 197202 (2022).

S6. Z. Q. Wang, *et al.*, Inverse Spin Hall Effect Dominated Spin-Charge Conversion in (101) and (110)-Oriented RuO_2 Films, Phys. Rev. Lett. **133**, 046701 (2024).

S7. A. Bose, *et al.*, Tilted spin current generated by the collinear antiferromagnet ruthenium dioxide, Nature Electronics **5**, 267 (2022).

S8. Y. Fan, *et al.*, Robust Magnetic-Field-Free Perpendicular Magnetization Switching by Manipulating Spin Polarization Direction in RuO_2/[Pt/Co/Pt] Heterojunctions, ACS Nano **18**, 26350 (2024).

S9. H.-L. Liang, *et al.*, Anisotropic spin-to-charge conversion in bismuth, Phys. Rev. B **106**, L201304 (2022).

S10. P.-H. Wu, *et al.*, Exploiting Spin Fluctuations for Enhanced Pure Spin Current, Phys. Rev. Lett. **128**, 227203 (2022).

S11. F.-J. Chang, *et al.*, Robust spin current generated by the spin Seebeck effect, Phys. Rev. Mater. **1**, 031401 (R) (2017).

S12. C. L. Bianchi, *et al.*, An XPS study on ruthenium compounds and catalysts, Mater. Chem. Phys. **29**, 297-306 (1991).

S13. Y. Kaga, *et. al.*, Ru and RuO_2 Thin Films by XPS, Surf. Sci. Spectra **6**, 68 (1999).

S14. C. D. Wagner, *et. al.*, in NIST X-ray Photoelectron Spectroscopy Database, Version 4.1, National Institute of Standards and Technology, Gaithersburg (2012).

S15. D. J. Morgan, *et. al.*, Resolving ruthenium: XPS studies of common ruthenium materials, Surf. Interface Anal. **47**, 1072–1079 (2015).

S16. D. Qu, *et al.*, Large enhancement of the spin Hall effect in Mn metal by Sn doping, Phys. Rev. Mater. **2**, 102001(R) (2018).
